# Multidrug-resistant *Aeromonas hydrophila* causing fatal bilateral necrotizing fasciitis in an immunocompromised patient: a case report

**DOI:** 10.1186/s13256-018-1854-1

**Published:** 2018-11-01

**Authors:** Alejandra Ugarte-Torres, Sarah Perry, Angela Franko, Deirdre L Church

**Affiliations:** 1 Department of Medicine, University of Calgary, 9-3535 Research Rd NW, Calgary, AB T2L 2K8 Canada; 2 Department of Pathology and Laboratory Medicine, University of Calgary, Calgary, Canada; 3 Department of Medicine, Snyder Institute for Chronic Diseases, Cummings School of Medicine, University of Calgary, Calgary, Canada

**Keywords:** Necrotizing fasciitis, Multidrug resistance, *Aeromonas hydrophila*

## Abstract

**Background:**

*Aeromonas hydrophila* is a water-dwelling, gram-negative rod-shaped bacterium, associated with diarrheal illness and, less commonly, necrotizing skin and soft tissue infections, especially among immunocompromised patients. Necrotizing fasciitis is associated with a high mortality rate, especially when caused by *Aeromonas spp*. Our patient was infected with an extremely aggressive form of multidrug-resistant *Aeromonas spp*. that produced both an extended-spectrum β-lactamase and an *Amp*C enzyme. Aeromonads are being recognized as important emerging pathogens because of their inherent antibiotic resistance profiles compounded by other virulence factors. These difficult-to-treat organisms can have significant implications in both clinical and public health settings.

**Case presentation:**

A 37-year-old Caucasian male with immunosuppression due to aplastic anemia being treated with cyclosporine, presented to hospital with relapsed disease. While in hospital, he subsequently developed overwhelming sepsis secondary to bilateral lower extremity necrotizing fasciitis. The necrotizing fasciitis was caused by a multidrug-resistant strain of *A. hydrophila.* Despite broad-spectrum antibiotics and aggressive surgical debridement, he succumbed to this severe invasive infection.

**Conclusions:**

Necrotizing fasciitis caused by *Aeromonas spp*. is a rare infection that may have a poor clinical outcome, particularly if the diagnosis is delayed and/or the organism is highly virulent and multidrug resistant. Enhanced education of clinicians and microbiologists is required to prevent unnecessary complications and improve survival.

## Background

*Aeromonas* species are gram-negative, non-sporulating, facultative, anaerobic small bacilli with a ubiquitous distribution [1]. They are water-dwelling, opportunistic pathogens commonly found not only in environmental sources such as fresh and brackish water, seafood, meat, and vegetables, but have also been isolated from chlorinated water, including hospital water supplies [2]. *Aeromonas spp.* infection has a variety of clinical presentations, including gastroenteritis, hepatobiliary tract infection, pneumonia, skin and soft tissue infections, empyema, meningitis, septic arthritis, osteomyelitis, endocarditis, and bacteremia [3]. Currently, there are more than 20 species identified, but only 7 have been recognized as human pathogens, namely *A. hydrophila, A. caviae, A. veronii biovar sobria, A. veronii biovar veronii, A. jandaei, A. trota,* and *A. schubertii*, with the first three being the most common [4]*.* Invasive *Aeromonas* infections usually occur amongst immunocompromised individuals, primarily in patients with solid or hematologic malignancies or hepatobiliary disease, but healthy individuals may also be affected after sustaining traumatic and crush injuries, near drowning events, and burns [2].

Necrotizing fasciitis (NF) has a rapid clinical progression that results in septic shock and associated multi-organ failure. Severe necrosis of the skin and underlying soft tissues is mediated by the release of bacterial toxins and proteases that lead to extensive inflammation [5, 6]. Some of the recognized risk factors associated with NF are immunosuppression, diabetes mellitus, alcoholism, end-stage renal disease, malignancy, chemotherapy, and previous surgery or trauma [7]. Polymicrobial NF, or type I, is the most common presentation (approximately 80% of cases), followed by monomicrobial, or type II (15% of cases), classically caused by group A *Streptococcus* [8]. Less than 5% of NF cases are caused by water-born organisms, most commonly *Vibrio vulnificus* and *Aeromonas spp.* Although the incidence of NF is low, estimated at 0.04 cases per 1000 persons-years in the United States [9], both types of NF are associated with a high mortality, ranging from 17 to 34% [1, 2], and cases caused by *Aeromonas spp*. have the highest reported mortality rate of up to 60% [10].

Herein, we report a case of a young immunocompromised male undergoing treatment for relapsed aplastic anemia that developed fatal sepsis secondary to bilateral leg NF caused by an *A. hydrophila* strain that was multidrug resistant*.*

## Case presentation

A 37-year-old Caucasian male with a known history of aplastic anemia (AA), presented to a rural hospital after a ground level fall. AA was diagnosed 10 months earlier after he was investigated for pancytopenia. A bone marrow biopsy showed cellularity of only 10% and the presence of a small paroxysmal nocturnal hemoglobinuria clone (less than 0.2%). He received standard combination treatment for AA with cyclosporine 225 mg orally twice daily, horse anti-thymocyte globulin (ATG) 40 mg/kg daily for 4 consecutive days, and prednisone 1 mg/kg daily. His other medications included daily Pantoloc 40 mg orally, daily Valtrex 500 mg orally, and daily Dapsone 50 mg orally for *Pneumocystis jirovecii* prophylaxis due to a reported allergy to trimethoprim/sulfamethoxazole. He had recently quit smoking and denied alcohol use but actively used other recreational drugs, including marijuana, cocaine, and methamphetamine. He was unemployed. He had no known other medical co-morbidities and was taking no other medications prior to developing AA. The etiology of AA was felt to be idiopathic because he had no improvement after an initial trial of sobriety. AA improved following immunosuppressive therapy and, although human leukocyte antigen typing was performed, a subsequent bone marrow transplant was deferred not only because of the medical therapeutic response but also due to his ongoing recreational drug use. Although he was no longer transfusion dependent a month after starting immunosuppressive therapy, his treatment compliance waned overtime due to regular ongoing recreational drug use of cocaine and methamphetamines. He routinely used unsterilized tap water for illicit drug injections, but he denied other exposure to fresh or salt water sources at home or in the community.

**Fig. 1:**
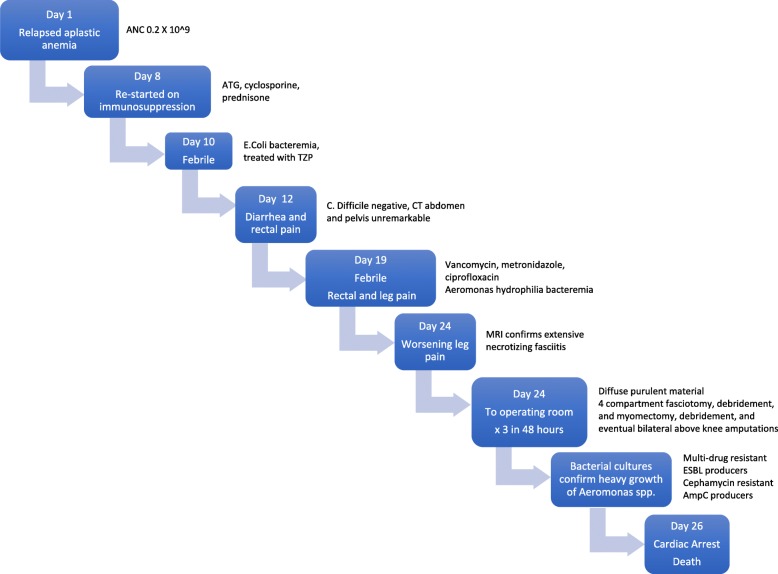
Timeline of patient’s course in hospital

On presentation to the emergency department he was not in distress, with a heart rate of 90 bpm and a blood pressure of 116/59. Severe pallor was noted upon examination, as well as a petechial rash and mild ecchymoses (Fig. 1). The rest of his physical assessment was normal, including a neurological examination. Admission bloodwork revealed severe pancytopenia with hemoglobin of 22 g/L, a platelet count of 1 × 10^9^/L, a white blood cell count of 3.7 × 10^9^/L, and an absolute neutrophil count of 0.2 × 10^9^/L (reticulocytes were not sent at admission, but 2 weeks into his hospitalization his absolute reticulocyte count was 12 × 10^9^/L with a reticulocyte percentage of 0.5). All other admission blood work was normal, including liver function tests (total bilirubin 9 μmol/L (reference < 21 μmol/L), alanine aminotransferase 13 μmol/L (reference < 41 μmol/L), alkaline phosphatase 66 U/L (reference 30–130 U/L)) and renal function tests (creatinine 63 μmol/L (reference 59–104 μmol/L), glomerular filtration rate 120 mL/min (reference < 59 mL/min)). He was stabilized and transferred to a tertiary care center where he was restarted on treatment for relapsed AA with a regimen that included cyclosporine (5 mg/kg/day) and prednisone 30 mg daily in addition to five doses of ATG. He remained transfusion dependent throughout his hospitalization.

On day 10 after admission, he developed generalized, mild (3/10), colicky abdominal pain with an associated fever > 38.5 °C. He was started empirically on piperacillin-tazobactam (PTZ) 3.375 gm intravenously every 6 hours. Two sets of blood cultures, each consisting of an anaerobic and aerobic BacT/Alert bottle (bioMérieux, Laval, Quebec), were collected peripherally and from his central line. *E. coli* grew in each bottle set at 10 and 11 hours, respectively. He then developed watery, non-bloody bowel movements, 3–4 times a day, associated with rectal pain. Real-time PCR for *Clostridium difficile* A/B toxin on a stool sample was negative. Computerized tomography of the abdomen and pelvis was also unremarkable. Repeat blood cultures were negative at 24 and 48 hours after the initial positive set. He improved dramatically after 7 days of intravenous PTZ and was stepped down to oral ciprofloxacin 500 mg orally twice daily to complete a further 7 days of therapy.

**Fig. 2:**
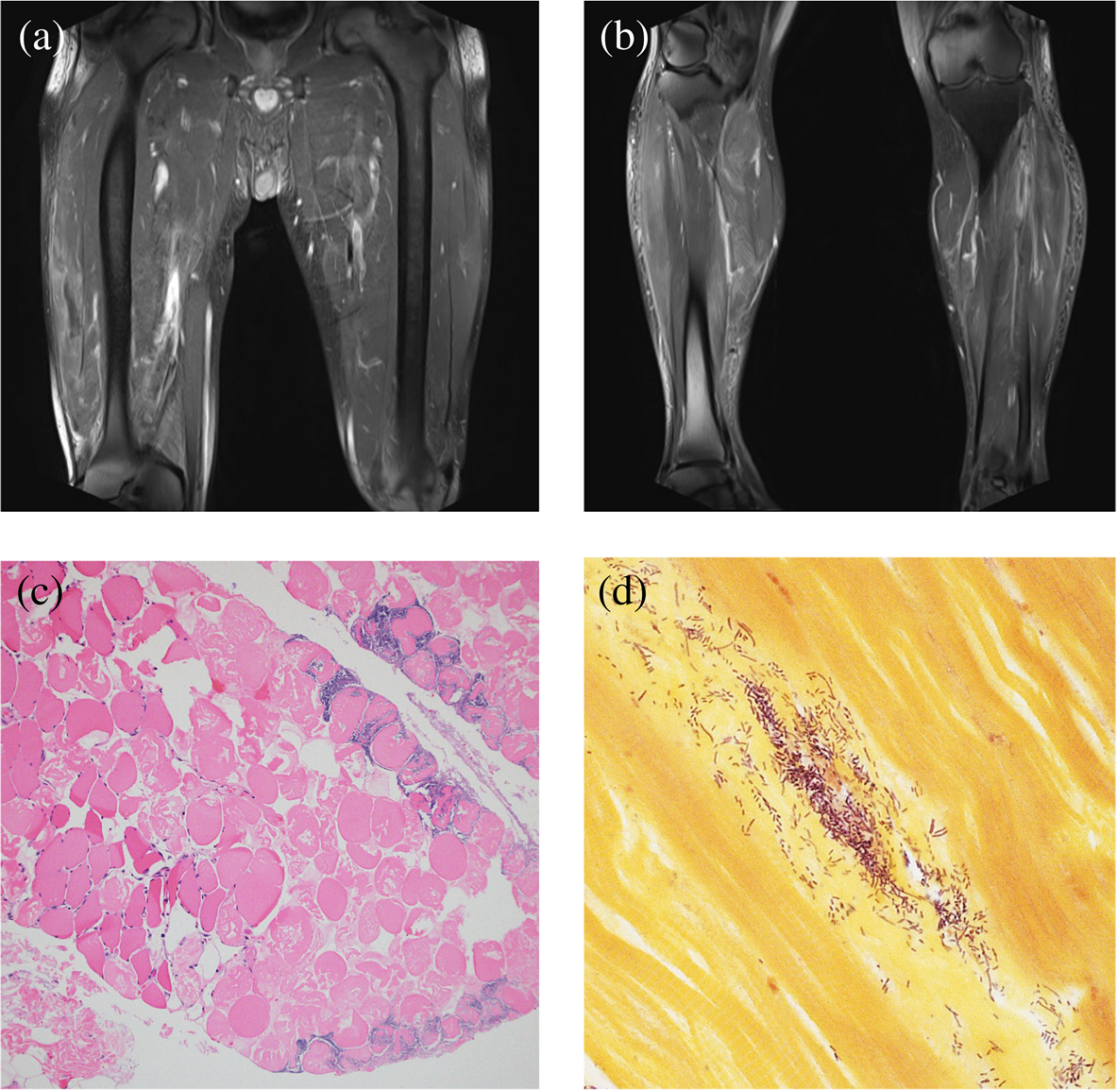
Coronal views of enhanced bilateral magnetic resonance imaging T2 image of hips (**a**) and legs (**b**). Muscular edema of vastus intermedius, vastus lateralis, mid rectus femoris, abductor magnus, abductor brevis, gracilis, and gastrocnemius associated with extensive multi-compartmental fascial edema bilaterally. No rim-enhancing collections or gas were noted. **c** Histologic image of necrotic muscle with associated loss of nuclei and striations. There are dense aggregations of bacteria infiltrating the muscle with no associated inflammatory response. Inset shows normal viable muscle for comparison. **d** Gram stain with numerous gram-negative bacilli, consistent with *Aeromonas hydrophila*

On day 19 of admission he developed acute continuous severe (9/10), non-radiating dull rectal pain, associated with a high-grade fever (40.4 °C). Vancomycin 1.5 g intravenously every 12 hours and metronidazole 500 mg orally twice daily were empirically started and ciprofloxacin was continued in the same dosage. Blood cultures that were collected from peripheral venipuncture and a peripherally inserted central catheter line grew *A. hydrophila* at 11 hours. The peripherally inserted central catheter line was immediately removed the next day (day 20 after admission). The same day he also began to complain of vague, mild, bilateral leg pain. Delayed serum sickness due to recent ATG administration was considered a possible cause for his new symptoms because clinical examination did not show erythema, edema, or deformities on either of his legs. However, sustained bacteremia was diagnosed by recovery of *A. hydrophila* from repeat blood cultures (i.e., one anaerobic and aerobic bottle set from two peripheral venipunctures) positive after 11 and 16 hours of incubation. Bilateral leg pain steadily worsened in intensity (10/10) over the next 48 hours, and the area of distribution of pain extended to the lateral aspect of the right thigh although physical examination remained unremarkable. Creatinine kinase was increased at 470 U/L (normal range for males, 0–195 U/L). Ultrasound venous Doppler of both legs also showed no evidence of deep venous thrombosis. However, magnetic resonance images of both legs showed extensive bilateral patchy multi-compartment muscular and fascial inflammatory changes highly concerning for NF (Fig. 2a, b).

Urgent initial surgical debridement was performed that evening. An extensive four-compartment fasciotomy, debridement, and myomectomy were performed on both legs. Extensive ‘dishwater’ purulent material was found in multiple compartments of both legs, including (1) the superficial posterior compartment between the gastrocnemius and soleus muscles, and (2) the lateral deep compartment. There was also clinical evidence of severe muscle necrosis of the tibialis anterior muscles in the anterior compartment of both legs. He was admitted to the Intensive Care Unit post-operatively. After consultation with the Infectious Diseases service and review of the antibiotic susceptibility profile of the previously isolated *A. hydrophila* strain, antibiotics were changed to meropenem 1000 mg intravenously every 8 hours and clindamycin 600 mg intravenously every 8 hours. High dose intravenous immunoglobulin (2 g/kg) was also given. All prior antibiotics were discontinued.

Gram stain of tissue samples from the right tibialis anterior muscle showed no neutrophils but that gram-negative bacilli were present, and subsequently grew a heavy amount of *A. hydrophila*. Gram stain and anaerobic culture from the right vastis lateralis muscle also did not show the presence of neutrophils or organisms but grew scant amounts of *A. hydrophila*. A genus-level identification as *Aeromonas* was obtained for all isolates from blood and tissue samples by matrix-assisted laser desorption ionization-time of flight (MALDI-TOF) mass spectrometry using a VITEK MS (bioMérieux, Laval, Quebec, Canada); since this technique has an accuracy of identification rate of 80–90% for species-level identification of *Aeromonas* [11], all isolates were also analyzed using in-house bi-directional 16S rRNA gene cycle sequencing of the V1-V3 (approximately first 500 bp), as previously described [12]. Broth microdilution susceptibility panel testing was performed and interpreted using published guidelines [13]. All isolates were multidrug resistant to ampicillin, ceftriaxone, ciprofloxacin, and trimethoprim/sulfamethoxazole but susceptible to meropenem and tetracycline. The isolates were confirmed to produce an extended-spectrum β-lactamase (ESBL) using published guidelines and the Mast Disc Test (Mast Group Ltd., Merseyside, UK) [13]. Production of an *Amp*C β-lactamase was shown by resistance to cefoxitin disk (30 μg) testing and the Mast Disc test (Mast Group Ltd.).

Two additional extensive surgical procedures for removal of necrotic tissue from both legs were undertaken in the next 24 hours. Bilateral above-knee amputations were performed during the last debridement as a life-saving measure because of extensive rapid progression of bilateral leg necrosis, and the patient’s rapid clinical deterioration with severe unremittent hemodynamic instability during the operation. Post-operatively, he required aggressive resuscitation for septic shock in the Intensive Care Unit with intractable hyperkalemia and severe acidosis, and anuric acute kidney failure (creatinine 210 μmol/L; normal range for males, 50–120 μmol/L). Despite all therapeutic interventions, the patient went into cardiac arrest and passed away within 2 hours after the final surgery.

Post-mortem examination at autopsy revealed findings related to the underlying AA, and evidence of septic shock secondary to extensive bilateral lower limb necrotizing myofasciitis. The bone marrow was markedly hypocellular and there was splenic enlargement at 331 g. The heart was enlarged (536 g). Cardiomegaly was likely a compensatory response to the AA due to the absence of atherosclerotic and hypertensive cardiovascular disease. In keeping with the patient’s severe septic shock, there was marked centrilobular necrosis of the liver, as well as petechial hemorrhages of the skin, heart, pleural surfaces, kidneys, and liver capsule. Histologic examination of skin and muscle from the left thigh showed necrosis of the muscle and deep subcutaneous adipose tissue, admixed with dense collections of gram-negative bacilli (Fig. 2c, d). However, in keeping with the AA, there was notably an absence of an acute inflammatory response.

## Discussion and conclusions

In summary, this 37-year-old male with AA developed rapidly progressive bilateral NF from *A. hydrophila* that produced both an ESBL and *Amp*C β-lactamase, and was confirmed to be multidrug resistant to multiple classes of antibiotics. Similar cases caused by multidrug-resistant strains of *A. hydrophila* have not previously been reported. The rapid clinical course in this severely immunocompromised man is also attributable to delayed diagnosis. This unique case demonstrates that physicians must have a high level of suspicion for necrotizing skin and soft tissue infection (NSSTI) in patients with increasing pain and creatinine kinase levels despite an unremarkable clinical examination.

NSSTIs caused by *Aeromonas spp.* infection are rare but are associated with very high mortality, reportedly between 60 and 75% in immunocompromised hosts [4]. NSSTI caused by *Aeromonas spp.* is the second most common clinical presentation caused by these organisms after diarrheal illness. Invasive *Aeromonas* infections have been reported in patients with underlying hematological conditions, including those with leukemia, lymphoma, and hematopoietic stem cell transplant recipients [14, 15]. Similar to our case, most of the previously reported NF cases have been associated with fatal septic shock and multi-organ failure [14, 16]. There is no history of preceding trauma or water exposure in 20–30% of patients with *Aeromonas*-related sepsis or NSSTI.

Patients who develop NSSTI from *Aeromonas* spp. are often found to have gastrointestinal colonization with this organism [17]. The pathogenesis of the severe NSSTI in our case most likely occurred by a similar mechanism given the preceding gastrointestinal symptoms and associated *E. coli* bacteremia. Although he was asymptomatic on admission, severe bilateral leg pain acutely developed shortly after the onset of gastrointestinal symptoms. Most likely, secondary endogenous bacteremia occurred due to bacterial gut translocation and resulted in the subsequent seeding in the soft tissues and muscles of both legs. Unfortunately, there was no stool culture performed to prove *Aeromonas* colonization. Although intestinal colonization with *Aeromona*s *spp.* in otherwise healthy individuals has been reported to be very low [18, 19], we surmise that this was the probable source in our patient given his severe immunosuppression. Alternatively, *A. hydrophila* infection may have been caused by the use of contaminated tap water for injection of recreational drugs while in hospital – something suspected but never objectively proven by his medical team. This mechanism is considered less likely because there were no other nosocomial infections due to *Aeromonas spp*. reported by Infection Prevention and Control surveillance data throughout the period of his admission.

*Aeromonas spp.* are relatively common in some ecologic niches, particularly salt or fresh water environments [2]. Environmental studies have demonstrated that these organisms do not routinely contaminate drinking and surface water samples or food, which leads to the ‘selection’ of specific pathogenic strains of *Aeromonas spp.* to produce invasive infection [20]. Pathogenic *Aeromonas spp.* have also been shown to produce several virulence factors including aerolysin-related cytotoxic enterotoxin*.* This endotoxin causes diarrhea and severe tissue damage by inducing apoptosis of host cells [21]. Multiple other proteins have also been linked to tissue damage, such as VIP-2, which not only has cytotoxic effects [22] but also inhibits bacterial phagocytosis. Two other mechanisms are associated with the pathogenesis of *Aeromonas* infections, namely its capacity to form biofilms and the high hemolytic activity of aerolysin-related cytotoxic enterotoxin [23]. *Aeromonas spp*. also produces several enzymes that have caseinase and elastase activity [24], which may also contribute to its ability to invade tissue and cause NF.

Finally, the multi-drug resistance profile of the *A. hydrophila* isolate in our patient was another major virulence factor that likely contributed to uncontrollable invasive infection and ultimate fatality in this case. On prior reports, drug resistance did not influence mortality [3], but none of the previously reported invasive *Aeromonas spp.* cases were caused by a highly multidrug-resistant strain as occurred herein. The major resistance mechanism reported to date for most Aeromonads is an inducible chromosomal β-lactamase [25, 26], including production of an ESBL and/or a metallo-β-lactamase active against carbapenems [27]. On a recent report from clinical isolates in France, only 2% were resistant to third-generation cephalosporins and 32% of isolates were resistant to carbapenems [28]. *Amp*C β-lactamase production by *Aeromonas spp.* is uncommon but has primarily been reported in isolates from environmental samples and stool samples from adults and children in India [29]. The most common metallo-β-lactamase produced by *Aeromonas spp.* is the CphA type, other types detected are VIM and IMP encoded on an integron and a plasmid, respectively [25, 30]. *Aeromonas* species were thought to be universally susceptible to fluoroquinolones [1], but the strain recovered from our case demonstrated high-level resistance to fluoroquinolones.

NF caused by *Aeromonas spp*. is rare but may result in high morbidity and mortality if a diagnosis is delayed and the organism is highly virulent and multidrug resistant, as demonstrated by our case. Enhanced education of clinicians and microbiologists is required to improve patient outcomes. Clinicians need to be aware of this organism’s potential to cause severe NSSTI, especially amongst immunocompromised patients or in normal hosts following traumatic injuries. Invasive NF infections due to *Aeromonas spp.* should be treated aggressively with surgical debridement and broad-spectrum antibiotics confirmed by laboratory testing to have documented susceptibility and clinical efficacy.

## Abbreviations

AA: aplastic anemia
ATG: anti-thymocyte globulin
ESBL: extended spectrum β-lactamase
NF: necrotizing fasciitis
NSSTI: necrotizing skin and soft tissue infections
